# Zn-Shik-PEG nanoparticles alleviate inflammation and multi-organ damage in sepsis

**DOI:** 10.1186/s12951-023-02224-3

**Published:** 2023-11-25

**Authors:** Jie Guo, Yuqing Miao, Fayi Nie, Fei Gao, Hua Li, Yuan Wang, Qi Liu, Tingbin Zhang, Xiaohang Yang, Li Liu, Haiming Fan, Qiang Wang, Haifa Qiao

**Affiliations:** 1 Shaanxi Collaborative Innovation Center of TCM Technologies and Devices, Shaanxi University of Chinese Medicine, Xianyang, 712046 China; 2 Shaanxi Key Laboratory of Integrated Acupuncture and Drugs, College of Acupuncture and Tuina, Shaanxi University of Chinese Medicine, Xianyang, 712046 China; 3 Center for Health Science and Engineering, School of Health Sciences and Biomedical Engineering, Hebei University of Technology, Tianjin, 300130 China; 4 Key Laboratory of Synthetic and Natural Functional Molecule Chemistry of the Ministry of Education, College of Chemistry and Materials Science, Northwest University, Xi’an, 710069 China

**Keywords:** Sepsis, Inflammatory, Shikonin, Reactive oxygen, Metal-polyphenol coordination

## Abstract

Sepsis is defined as a life-threatening organ dysfunction caused by excessive formation of reactive oxygen species (ROS) and dysregulated inflammatory response. Previous studies have reported that shikonin (Shik) possess prominent anti-inflammatory and antioxidant effects and holds promise as a potential therapeutic drug for sepsis. However, the poor water solubility and the relatively high toxicity of shikonin hamper its clinical application. To address this challenge, we constructed Zn^2+^-shikonin nanoparticles, hereafter Zn-Shik-PEG NPs, based on an organic-inorganic hybridization strategy of metal-polyphenol coordination to improve the aqueous solubility and biosafety of shikonin. Mechanistic studies suggest that Zn-Shik-PEG NPs could effectively clear intracellular ROS via regulating the Nrf2/HO-1 pathway, meanwhile Zn-Shik-PEG NPs could inhibit NLRP3 inflammasome-mediated activation of inflammation and apoptosis by regulating the AMPK/SIRT1 pathway. As a result, the Zn-Shik-PEG NPs demonstrated excellent therapeutic efficacies in lipopolysaccharide (LPS) as well as cecal ligation puncture (CLP) induced sepsis model. These findings suggest that Zn-Shik-PEG NPs may have therapeutic potential for the treatment of other ROS-associated and inflammatory diseases.

**Supplementary Information:**

The online version contains supplementary material available at 10.1186/s12951-023-02224-3.

## Introduction

Sepsis, a highly lethal syndrome resulting from a dysregulated host response to infection, is a major global health problem [1, 2]. Despite recent treatment advances, the overall mortality rate of sepsis remains unexpectedly high and sepsis remains the leading cause of mortality in the intensive care unit [3]. Currently, the main therapeutic strategies are intensively dependent on broad-spectrum antibiotic therapy or fluid resuscitation, which have limited efficacy and inevitably associated with severe side effects [4]. Accordingly, there is a pressing need to develop innovative and effective strategies for sepsis therapy. Pathologically, sepsis is characterized by over-exuberant inflammation and overproduction of reactive oxygen species (ROS), which can induce significant cell and organ injury and lead to multiple organ failure syndrome eventually [5, 6]. Thus, seeking an effective method which can exert anti-oxidant and anti-inflammatory effects simultaneously is significant to manage sepsis.

Traditional herbal medicine, a gift from nature, have magical functions in the treatment of sepsis [7, 8]. As a component of Chinese herbal medicine, shikonin, also known as “Zi-cao”, possesses remarkable pharmacological activities such as anti-inflammation, anti-cancer, anti-oxidant and so on [9, 10]. For instance, shikonin conferred significant protection against sepsis by attenuating release of proinflammatory cytokines [11]. Moreover, it was reported that shikonin also maintained broad spectrum of ROS scavenging activities [12]. However, shikonin exhibits low water solubility, low bioavailability and high toxicity, which may result in insufficient therapeutic benefits and limit its further clinical application [13].

A metal-polyphenol coordination-based organic-inorganic hybridization strategy getting widely used to improve the bioavailability of traditional Chinese medicines recently [14–17]. For example, Fe-curcumin nanoparticles can synergistically scavenge ROS and suppress inflammation for the treatment of acute lung injury [18]. Cu_2 − x_Se-PVP-Qe nanoparticles fabricated by using copper ions coordinated with quercetin have multi-enzyme-like activity and can effectively eliminate ROS in the brain [19]. Therefore, we hypothesize that the organic-inorganic hybrid strategy based on metal polyphenol coordination may overcome the deficiencies of shikonin and can exert more prominent anti-inflammatory and ant-oxidant effects when employed in sepsis treatment.

**Scheme 1:**
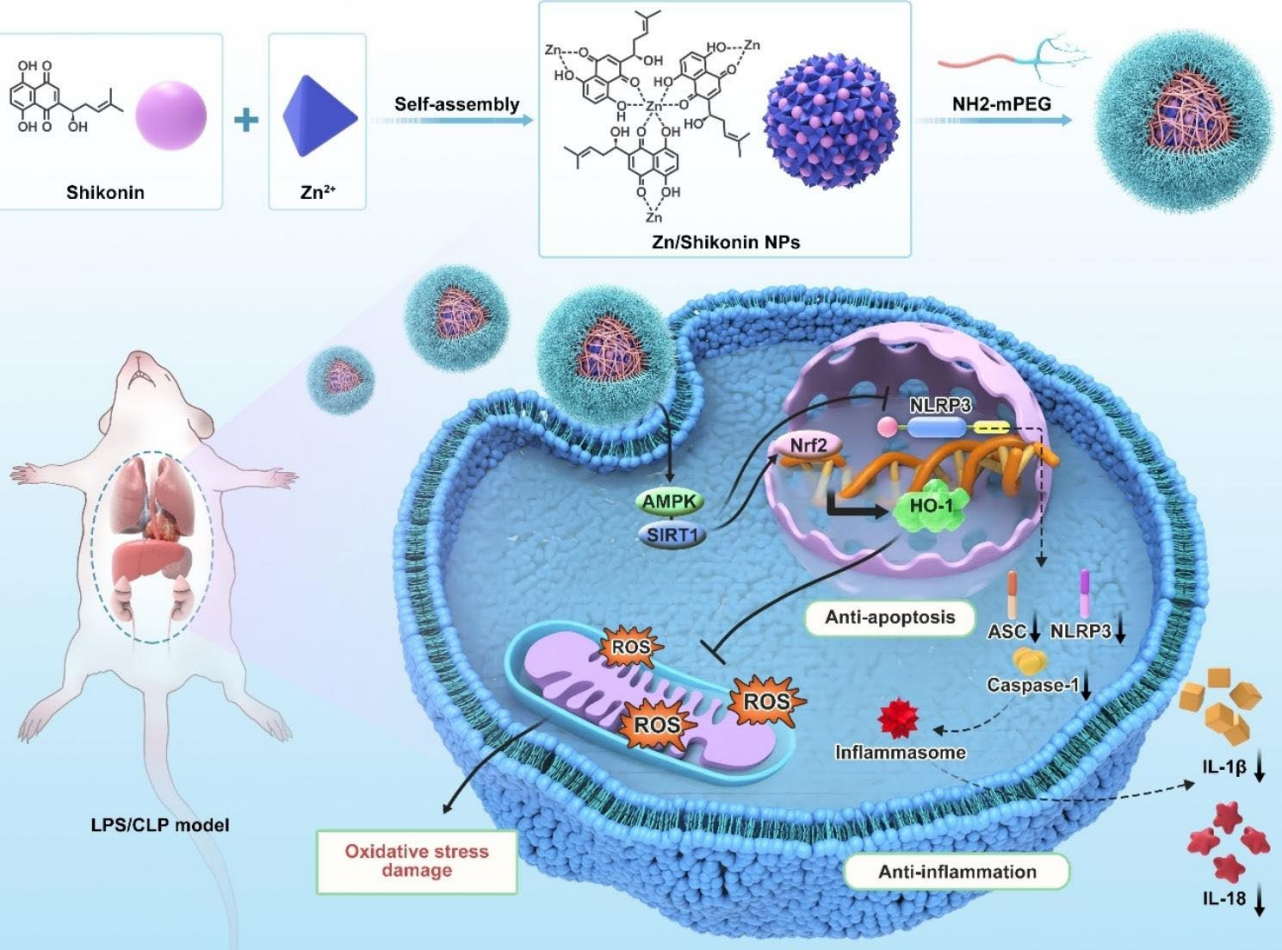
Schematic illustration of Zn-Shik-PEG NPs preparation and the ant-oxidant and anti-inflammation effects of Zn-Shik-PEG NPs in sepsis

Herein, we developed Zn-shikonin-PEG hybrid nanoparticles (Zn-Shik-PEG NPs) by means of metal-polyphenol coordination between Zn^2+^ and shikonin to achieve ROS scavenging and anti-inflammation for improving sepsis treatment (Scheme 1). Zinc is an indispensable trace element in human metabolism and was closely related to anti-inflammatory effects [20, 21]. PEG was used to confer high biocompatibility and physiological stability to the nanoparticles [22]. Therefore, as-prepared Zn-Shik-PEG NPs is expected to possess good biosafety and biocompatibility. We further conducted meticulous characterizations and experiments in vitro and in vivo to verify their potential therapeutic effects on sepsis animal models.

## Results and discussion

### Preparation and characterization of the Zn-Shik-PEG NPs

**Fig. 1:**
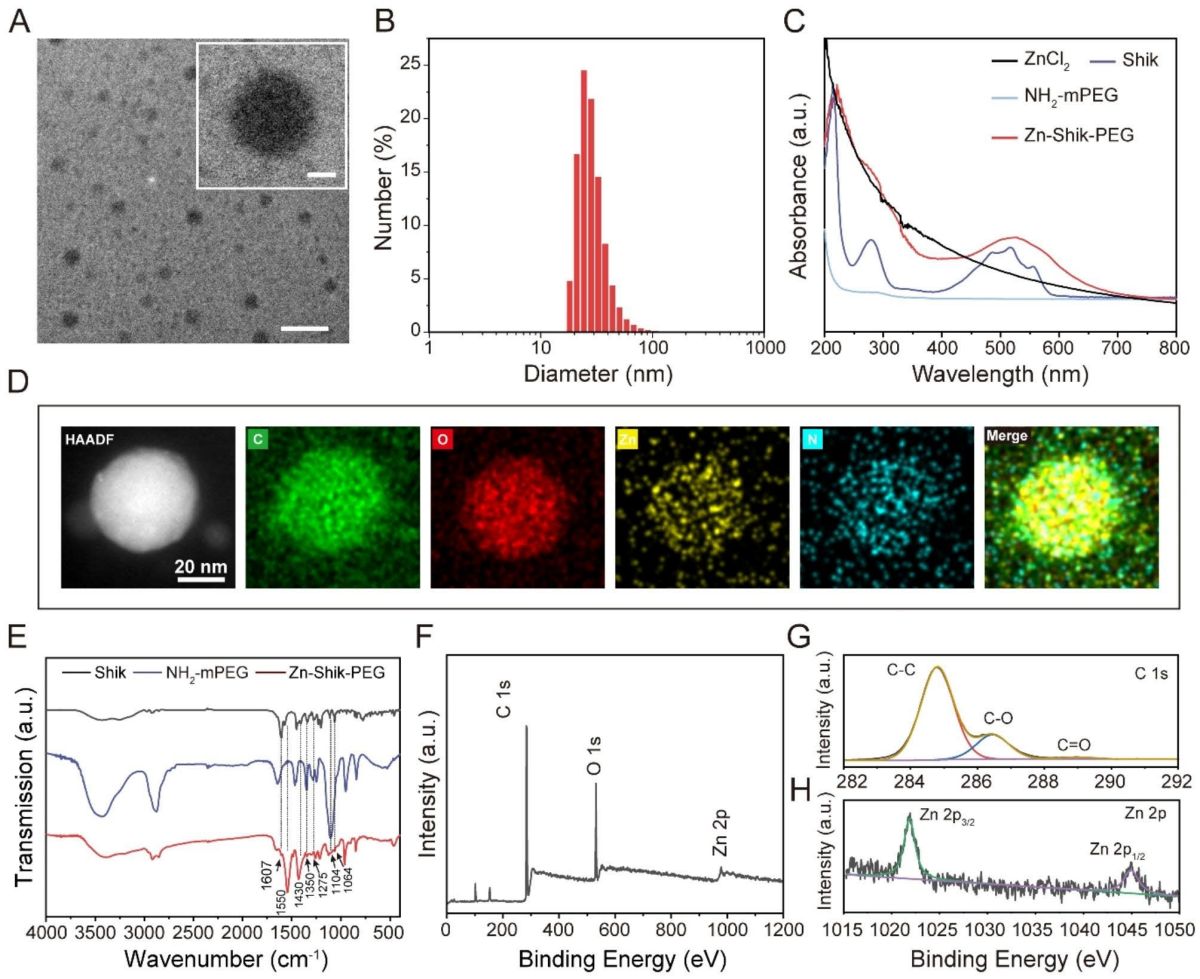
Preparation and characterization of Zn-Shik-PEG NPs. (**A**) TEM image of Zn-Shik-PEG NPs. Scale bar is 100 nm (inset: enlarged image of a single nanoparticle, scale bar: 10 nm). (**B**) Hydrodynamic size distribution of Zn-Shik-PEG NPs. (**C**) UV–vis absorption spectra of Shik, NH_2_-mPEG and Zn-Shik-PEG. (**D**) Energy dispersion spectroscopy (EDS) mapping images of Zn-Shik-PEG NPs. (**E**) FTIR spectra of Zn-Shik-PEG NPs. (F − H) Full scan XPS survey spectrum (**F**) and the high-resolution spectra of Zn-Shik-PEG for C 1s (**G**) and Zn 2p (**H**)

Zn-Shik NPs were formed by mixing ZnCl_2_ aqueous solutions and shikonin (dispersed in ethanol) simply. The phenolic hydroxyl of shikonin allows it to rapidly self-assembled with zinc ions, resulting in a three-dimensional network. Upon adding shikonin dropwise to zinc chloride solution, the solution changed from colorless to blue, which indicating that the metal ions rapidly coordinated with the phenolic hydroxyl groups of shikonin [13, 18]. The as-prepared Zn-Shik nanoparticles were then conjugated with the NH_2_-mPEG via Schiff-Base reactions, yielding Zn-Shik-PEG nanoparticles. Transmission electron microscopy (TEM) showed that the Zn-Shik-PEG NPs have a uniform size of ≈ 30 nm (Fig. 1A), which was well consistent with the dynamic light scattering (DLS) measurement (Fig. 1B). Additionally, PEGylation did not significantly alter the size or morphology of the Zn-Shik NPs (Figure S1). EDS mapping show that C, O, Zn elements are distributed throughout the nanoparticle (Fig. 1D). Furthermore, there were no obvious size changes of Zn-Shik-PEG nanoparticles in PBS over one week, indicating the good colloidal stability of Zn-Shik-PEG NPs (Figure S2). The UV − vis absorption spectrum shows that characteristic absorption peaks of Zn-Shik-PEG NPs were close to shikonin (Fig. 1C), indicating the coordination between zinc ions and shikonin. Fourier-transform infrared (FTIR) spectra of the Zn-Shik-PEG nanoparticles further indicate that the reaction between phenolic hydroxyl of shikonin and Zn^2+^, as evidenced by the lowered intensity of HO-C stretching peak around 1275 cm^− 1^ (Fig. 1E, Table S3) [23]. In addition, the X-ray photoelectron spectroscopy (XPS) survey scan spectra clearly showed the presence of C, O, and Zn elements (Fig. 1F), which consistent with the EDS elemental mapping. The C 1s spectra were deconvoluted into three peaks. the peaks at 284.8, 286.4, and 289.0 eV can be ascribed to C-C, C-O, and C = O bonds, respectively (Fig. 1G) [24]. Figure 1 H show the Zn 2p high resolution XPS spectrum. The two characteristics peaks at 1044.9 and 1021.9 eV can be assigned to Zn 2p_1/2_ and 2p_3/2_, respectively [25]. The above results indicated the successful formation of Zn-Shik-PEG nanoparticles.

### ROS scavenging via Nrf2/HO-1 pathway of Zn-Shik-PEG NPs in Vitro

ROS has been implicated as a pivotal factor in the pathophysiology of sepsis. During the progression of sepsis, increased ROS production and/or decreased ROS scavenging capacity significantly contribute to immune cell dysfunction. Accordingly, scavenging excess ROS holds promise for the treatment of sepsis [26, 27]. Since shikonin is a well-known natural polyphenol that has been spotlighted as an ROS scavenger, we tested the ROS scavenging capabilities of Zn-Shik-PEG NPs. We first studied the cytotoxicity of Zn ions, PEG, shikonin and the as-synthesized Zn-Shik-PEG NPs against Raw 264.7 macrophages (Figure S3). We found that shikonin at the concentrations greater than 5 µg/mL effectively suppressed cell growth, while Zn ions, PEG and Zn-Shik-PEG did not exhibit significant cytotoxicity at tested concentrations. The in vitro cytotoxicity results suggested that shikonin cytotoxicity can be reduced by the Zn-Shik-PEG NPs. Furthermore, following treatment with the indicated dose of shikonin or Zn-Shik-PEG NPs, RAW264.7 cells were stimulated with lipopolysaccharide (LPS) to evaluate their anti-inflammatory effects. As shown in Figure S4, LPS significantly decreased the cell viability to 13%, and the cell viability was increased by 20%~48% with different concentrations of shikonin (1, 3, 5 µg/ml) pretreatment, while pretreatment with different concentrations of Zn-Shik-PEG NPs (2, 5, 10 µg/ml) increased the cell viability up to 59%~86%. This result demonstrates that Zn-Shik-PEG NPs can significantly improve the cell viability compared with shikonin. Moreover, when the concentration of Zn-Shik-PEG NPs was 5 µg/mL, the cell viability was significantly increased when compared with control. Therefore, 5 µg/mL of Zn-Shik-PEG NPs was selected as an optimal concentration for subsequent experiments.

**Fig. 2:**
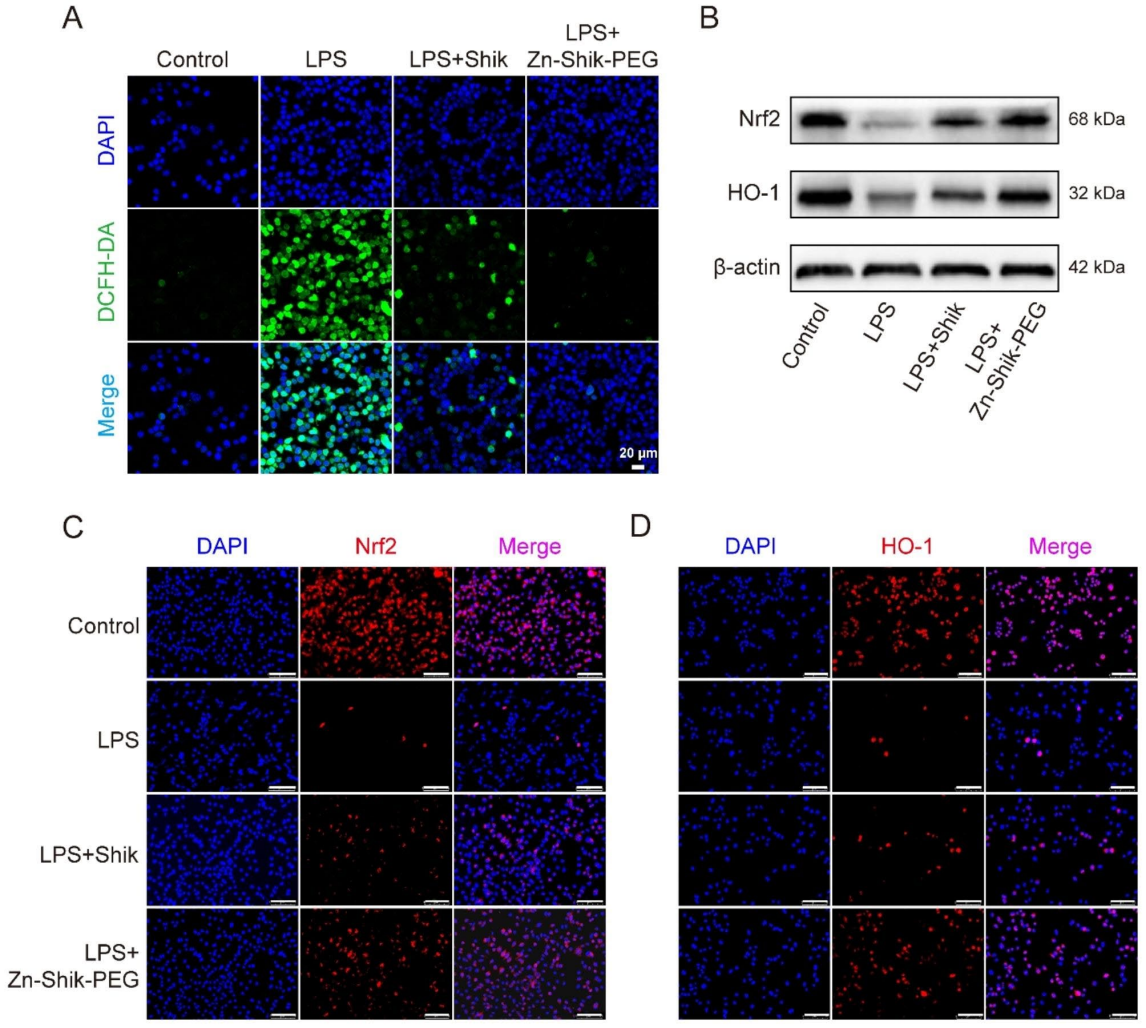
ROS scavenging via Nrf2/HO-1 pathway of Zn-Shik-PEG NPs in vitro. (**A**) Intracellular ROS imaging in RAW264.7 macrophages. (**B**) Representative western blotting bands of Nrf2, HO-1. (**C** and **D**) Representative immunofluorescence images of Nrf2 and HO-1 in RAW264.7 cells after different treatments. (Nucleus: blue; Nrf2 and HO-1: red). Scale bar: 75 μm

Next, the scavenging activities of the Zn-Shik-PEG NPs for intracellular ROS were measured in LPS-induced RAW264.7 cells. As shown in Fig. 2A and Figure S5, an excessive amount of ROS produced after LPS treatment, as evidenced by increased green fluorescence. When the cells were pretreated with shikonin or Zn-Shik-PEG NPs, the mean fluorescence intensity of the cells markedly weakened, suggesting the general intracellular ROS scavenging capability. Meanwhile, we could notice that the Zn-Shik-PEG NPs can more effectively eliminate intracellular ROS compared with the shikonin group, and this is likely due to Zn-Shik-PEG NPs improved water solubility and bioavailability of shikonin. In addition, nuclear factor erythroid 2-related factor (Nrf2), a major regulator of the cellular defense system against oxidative stress, binds with antioxidant response elements (AREs) to initiates transcription of cytoprotective heme oxygenase-1 (HO-1) under oxidative stress conditions [28, 29]. As a result, Nrf2-induced antioxidant kinases can scavenge excess ROS and suppress excessive inflammatory responses [5]. Therefore, here, western blot analysis and immunofluorescence staining were employed to explore the effect of Zn-Shik-PEG NPs on the Nrf2 and HO-1 expression levels in vitro. As shown in Fig. 2B and Figure S6, LPS-mediated reduction of the Nrf2 and HO-1 proteins was significantly suppressed by Zn-Shik-PEG NPs, demonstrating that Zn-Shik-PEG NPs might enhance the endogenous antioxidant defense to counteract LPS-induced oxidative stress. Immunofluorescence staining analysis further supported the results of Western blotting (Fig. 2C, D and Figure S7). After LPS stimulation, the expression of Nrf2 and HO-1 were significantly reduced compared with control condition (*P* < 0.01). Shikonin treatment could slightly increase the expression of both proteins, whereas the Zn-Shik-PEG NPs group could enhance the expression of NRF2 and HO-1 to a greater degree. Collectively, these results suggest that Zn-Shik-PEG NPs could decrease the LPS-induced ROS via Nrf2/HO-1 pathway.

### Anti-inflammation and anti-apoptosis effect of Zn-Shik-PEG NPs in LPS-Stimulated macrophage cells

**Fig. 3:**
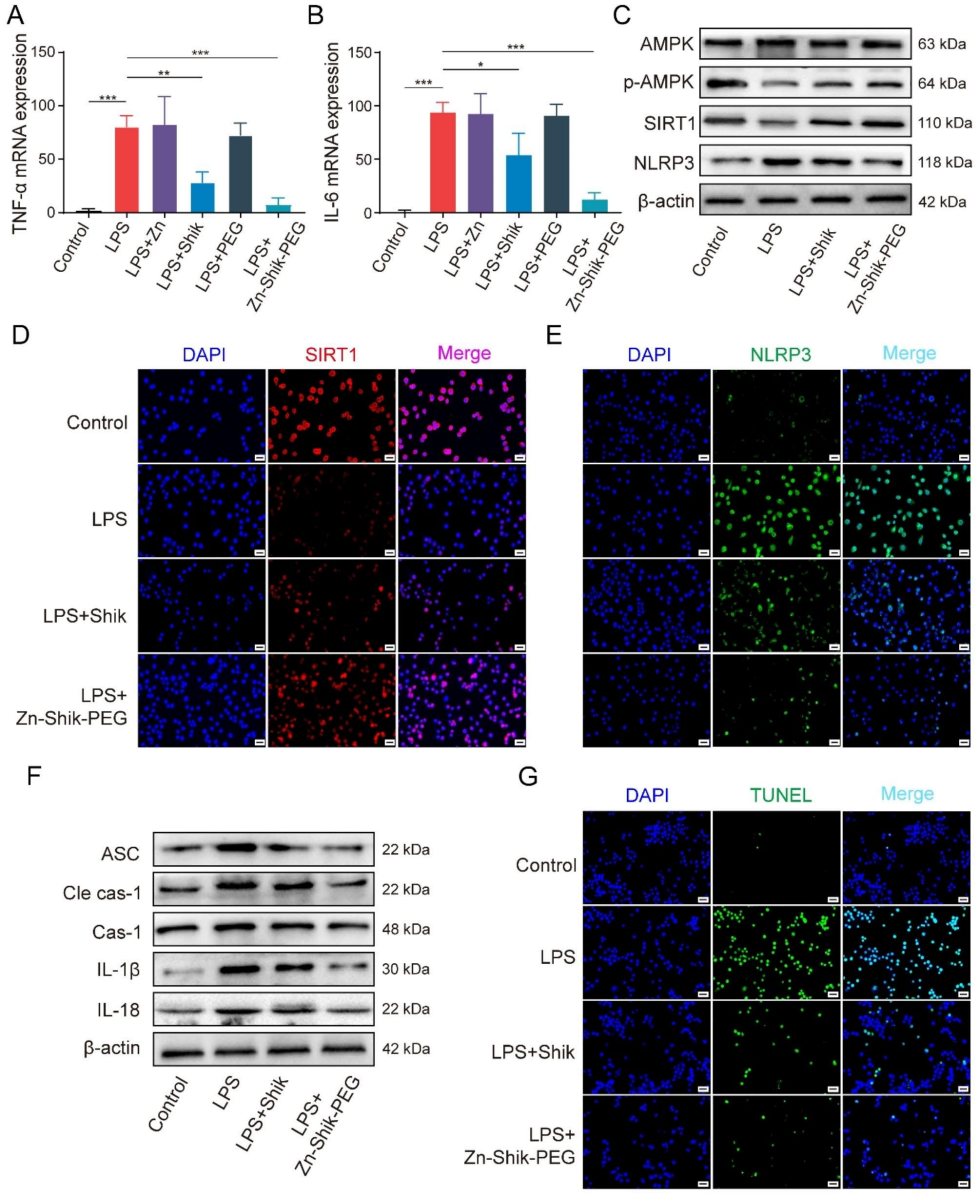
Modulation of the AMPK/SIRT1/NLRP3 inflammatory signaling pathway. (**A** and **B**) RT-qPCR analysis of the expression of TNF-α and IL-6. (**C**) Representative western blotting bands of AMPK, p-AMPK, SIRT1, NLRP3. (**D** and **E**) Representative immunofluorescence images of SIRT1 and NLRP3 in RAW264.7 cells after different treatments. (Nucleus: blue; SIRT1: red; NLRP3: green). (**F**) Representative western blotting bands of ASC, cleaved caspase-1, caspase-1, IL-1β, IL-18. (**G**) Representative TUNEL staining from different treatment of RAW264.7 cells. (Nucleus: blue; TUNEL: green). Scale bar: 20 μm. *p < 0.05, ** *p* < 0.01, and ***p < 0.001

Previous studies have found increased proinflammatory cytokines, such as TNF-α and IL-6, in patients with sepsis [30]. A protective strategy for blocking or eliminating these cytokines has been proposed as a valid treatment for sepsis [31]. To evaluate the anti-inflammatory effects of Zn-Shik-PEG NPs, we applied RT-qPCR to detect the mRNA expression levels of pro-inflammatory cytokines in LPS-stimulated RAW264.7 cells. As shown in Fig. 3A, B, LPS stimulation markedly increased the expression levels of TNF-α, IL-6, while pretreatment with Zn-Shik-PEG NPs effectively decreased their expression, which was close to the blank control. Regarding shikonin treatment groups, the increase of related genes also showed somewhat inhibition, whereas Zn ions and PEG does not affect TNF-α or IL-6 expression. These results indicated that the anti-inflammation activity of Zn-Shik-PEG was mainly derived from shikonin.

Adenosine monophosphate-activated protein kinase (AMPK) plays an important regulatory role in the inflammatory response during sepsis [32]. Accumulating evidence indicated that shikonin may exert anti-inflammatory effects by activating AMPK [11, 33]. On the other hand, NLRP3 inflammasome is also a critical signaling mediator of inflammatory damage in sepsis, and SIRT1 plays an important role in the regulation of NLRP3 inflammasome [34]. Furthermore, several studies have suggested a relationship between SIRT1 and the AMPK signaling pathway [35]. Based on the above analysis, we hypothesized that the excellent anti-inflammatory effect of Zn-Shik-PEG NPs might be through the activation of AMPK-SIRT1 signaling pathway to inhibit NLRP3 inflammasome activation in LPS-induced RAW264.7 cells. To verify this hypothesis, we analyzed the total AMPK expression, phosphorylated AMPK (p-AMPK), SIRT1 and NLRP3 by western blotting assay and immunofluorescence assay. As shown in Fig. 3C and Figure S8, LPS stimulation significantly reduced the expression of phosphorylated AMPK (p-AMPK) and SIRT1, while it increased the expression of NLRP3, and which were partly reversed by Zn-Shik-PEG NPs. Similar results were observed in immunofluorescence assay, Zn-Shik-PEG NPs increased the expression level of SIRT1, and reduced NLRP3 expression (Fig. 3D, E and Figure S9). To further confirm whether Zn-Shik-PEG NPs can reduce LPS-stimulated cellular inflammatory damage through AMPK activation, we treated cells with BML-275, an AMPK inhibitor [36]. As shown in Figure S10, Zn-Shik-PEG NPs could indeed reverse the inhibitory effect of BML-275, thus improving p-AMPK and SIRT1 expression, while reducing NLRP3 expression. These results suggest that Zn-Shik-PEG NPs attenuated LPS induced inflammatory damage by activating the AMPK/SIRT1 signaling pathway.

As we know, NLRP3 inflammasomes are multimeric complexes composed of NLRP3, apoptosis-associated spot-like protein (ASC), and cystatin-1 precursor (pro-caspase 1). In response to danger signals, NLRP3 recruits ASC and pro-caspase 1, leading to cleavage of pro-caspase 1 to active caspase 1 (Cas-1), which further promotes maturation and release of pro-inflammatory cytokines (e.g., IL-1β and IL-18), consequently triggering downstream inflammatory responses [37, 38]. Therefore, we next checked the expression levels of ASC, caspase 1 and cleavage caspase 1 (Cle cas-1). As shown in Fig. 3F and Figure S11, ASC, cleaved caspase-1 were upregulated after LPS stimulation, whereas Zn-Shik-PEG NPs attenuated these effects. As a result, Zn-Shik-PEG NPs could reduce the expression of IL-1β, IL-18 in RAW264.7 after LPS stimulation. Moreover, the NLRP3 inflammasome-mediated caspase-1 activation and IL-1β and IL-18 release are closely associated with apoptosis [39, 40]. Therefore, we performed TUNEL staining as further evidence of apoptosis. As shown in Fig. 3G and Figure S12, LPS stimulation significantly increased apoptosis in RAW264.7 cells, but Zn-Shik-PEG NPs can reduce its apoptosis. Taken together, Zn-Shik-PEG NPs could activate AMPK/SIRT1 signaling pathway, decrease NLRP3-related proteins expression, as well as inhibited apoptosis, thus reduced the release of inflammatory factors.

### Anti-inflammatory therapeutic activity of Zn-Shik-PEG NPs in Sepsis mice

**Fig. 4:**
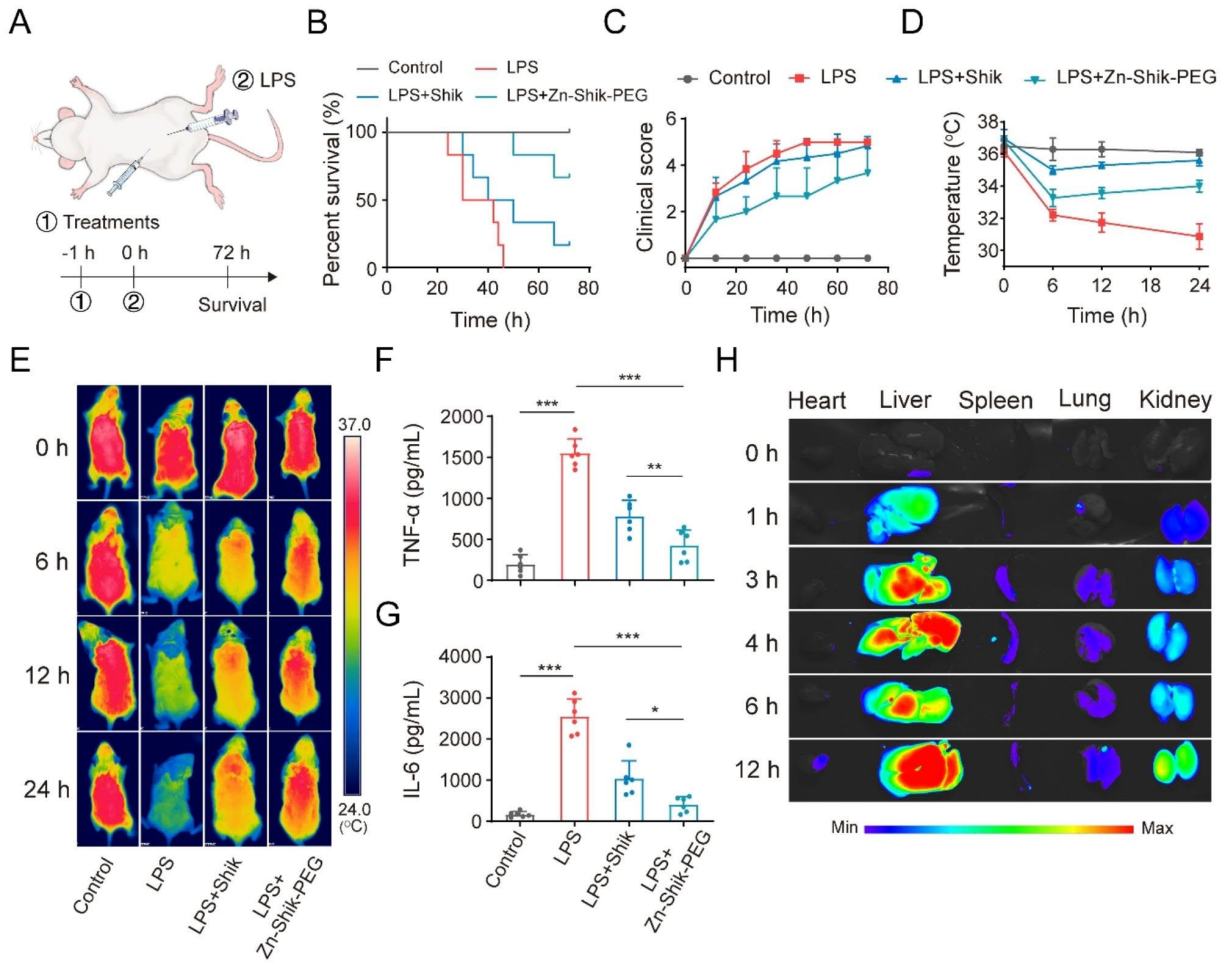
Therapeutic efficacy of the Zn-Shik-PEG NPs against LPS-induced sepsis in mice. (**A**) Experimental procedures for the LPS-induced sepsis mouse model. (**B**, **C**) Survival and clinical score analysis of the mice. (**D**, **E**) Body temperature change and thermographic images of mice in different treatment groups. (**F**, **G**) The level of pro-inflammatory cytokine TNF-α (**F**) and IL-6 (**G**) in the serum of septic mice. (**H**) Distribution of Cy5.5-labelled Zn-Shik-PEG NPs in main organs. Data are presented as mean ± SD (n = 3, 6). *p < 0.05, ** *p* < 0.01, and ***p < 0.001

Next, we used well-established models of LPS injection-induced endotoxemia to explore the in vivo performance of Zn-Shik-PEG NPs. Animals received a single intraperitoneal injection (i.p.) with Zn-Shik-PEG NPs, followed by a single i.p. of LPS (Fig. 4A) (10 mg/kg body weight, 1 h apart). The survival rate, clinical score (clinical score criteria are listed in the Supplementary Table S2), body temperature of mice were recorded. As shown in Fig. 4B, all mice in the LPS group died within 48 h. The shikonin treated group showed a slight improvement at 72 h (17%) while Zn-Shik-PEG NPs delayed mouse death and even rescued more than half of the mice within 72 h after LPS induction (67%), which supporting the protective effect of Zn-Shik-PEG NPs in the acute septic toxicity model. A significantly reduced clinical score was observed in the Zn-Shik-PEG NPs treatment groups (Fig. 4C), demonstrating restoration of physical state after treatment. The changes of body temperature in mice are essential features of sepsis, giving an indication of the gravity of the disease and the impairment of immune response upon infection [24, 41]. Thus, we also monitored body temperature of the mice. As shown in Fig. 4D, E, LPS induced a significant decrease in temperature (from 36.2 to 30.9 °C) within 24 h. while Zn-Shik-PEG NPs showed a strong protective effect on the degree of temperature decrease in mice, demonstrating the beneficial effects of Zn-Shik-PEG NPs in reversing sepsis-associated hypothermia. We next examined the therapeutic efficacy of Zn-Shik-PEG NPs using a cecal ligation and puncture (CLP)-induced sepsis model (Figure S13A). Zn-Shik-PEG NPs recovered body temperature, diminished clinical score, and improved survival in CLP mice (Figure S13B-E). To further evaluate the therapeutic efficacy of the Zn-Shik-PEG NPs, the proinflammatory cytokines TNF-α and IL-6, which are predominantly secreted by activated immune cells, were investigated (Fig. 4F, G and Figure S13F, G). Large quantities of TNF-α and IL-6 were detected in the serum of sepsis model group (either LPS or CLP induced model). When treated with Zn-Shik-PEG NPs, the level of inflammatory cytokines dropped significantly, which is consistent with the results of in vitro experiment.

Afterwards, we assessed the biodistribution of Zn-Shik-PEG NPs in LPS-treated mice. Mice were injected (i.p.) with Cy5.5-labelled NPs and killed at different time points for ex vivo fluorescence imaging. As shown in Fig. 4H, Cy5.5-labelled Zn-Shik-PEG NPs can reach several organs in mice including liver, kidney, lung, spleen, which are the most vulnerable target organs in sepsis. Zn-Shik-PEG NPs showed long retention in these organs, allowing for long-term protection against multi-organ damage in mice.

**Fig. 5:**
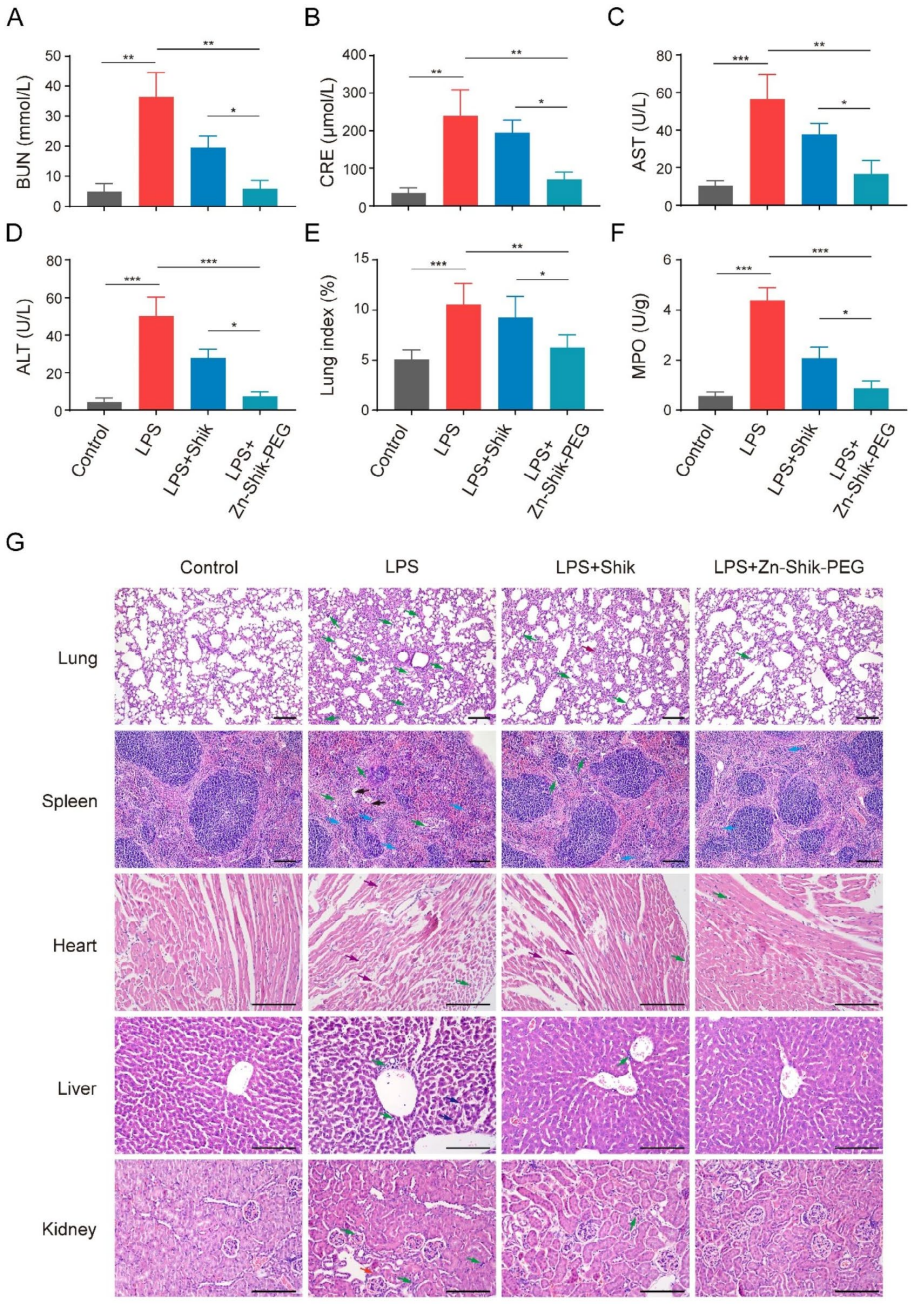
Protective effect of Zn-Shik-PEG NPs on LPS-induced multi-organ damage. (**A**-**D**) BUN, CRE, AST, ALT, levels in serum were detected by ELISA kits. (**E**) Lung index in each treatment group. (**F**) MPO activity was measured in lungs obtained 24 h after i.p. of LPS. (**G**) H&E staining of lung, spleen, heart, liver, kidney tissue. Green arrows indicate inflammatory cell infiltration; dark red arrow indicates interstitial thickening; black arrows indicate nuclear pyknosis; blue arrows indicate megakaryocytes; purple arrows indicate myocardial fiber breakage; dark blue arrows indicate hepatocellular necrosis; red arrow indicates renal tubule epithelial cell edema. Scale bar: 50 μm. Data are presented as mean ± SD (n = 3, 6). *p < 0.05, **p < 0.01, and ***p < 0.001

To investigate the NPs’ protective effect on multi-organ damage in sepsis mice, we performed biochemical and histopathological analysis. Serum biochemical analysis showed that urea nitrogen (BUN), and creatinine (CRE) (renal function indicators) levels and alanine transferase (ALT) and aspartate aminotransferase (AST) (liver function indicators) levels were significantly increased in LPS-treated group. In contrast, Zn-Shik-PEG NPs could significantly reduce the expression of the above markers (Fig. 5A-D). Furthermore, we also observed severe sepsis-induced lung injury in LPS mice, and the increased severity of lung injury was evidenced by increased lung index as well as myeloperoxidase (MPO) activity levels in the lung of LPS mice, and which can be reduced by Zn-Shik-PEG NPs (Fig. 5E, F). Similar results were obtained in CLP-induced sepsis model (Figure S14A-F). Consistently, hematoxylin-eosin staining (H&E staining) analysis validated the protective effects of the NPs in preventing major organ failure. As shown in Fig. 5G and Figure S14G, the organs of septic mice showed significant pathologic changes, such as inflammatory cell infiltration, alveolar wall thickening, the number of megakaryocytes increased, myocardial fiber breakage, hepatocellular necrosis, tubular epithelial cell edema. In contrast, the degree of organ damage was effectively reversed after Zn-Shik-PEG administration. Semiquantitative assessment using inflammation score also showed similar results, which the score was significantly lowered when septic mice (both LPS and CLP induced model) received Zn-Shik-PEG administration (Figure S15).

### Mechanism of action of Zn-Shik-PEG NPs in vivo

**Fig. 6:**
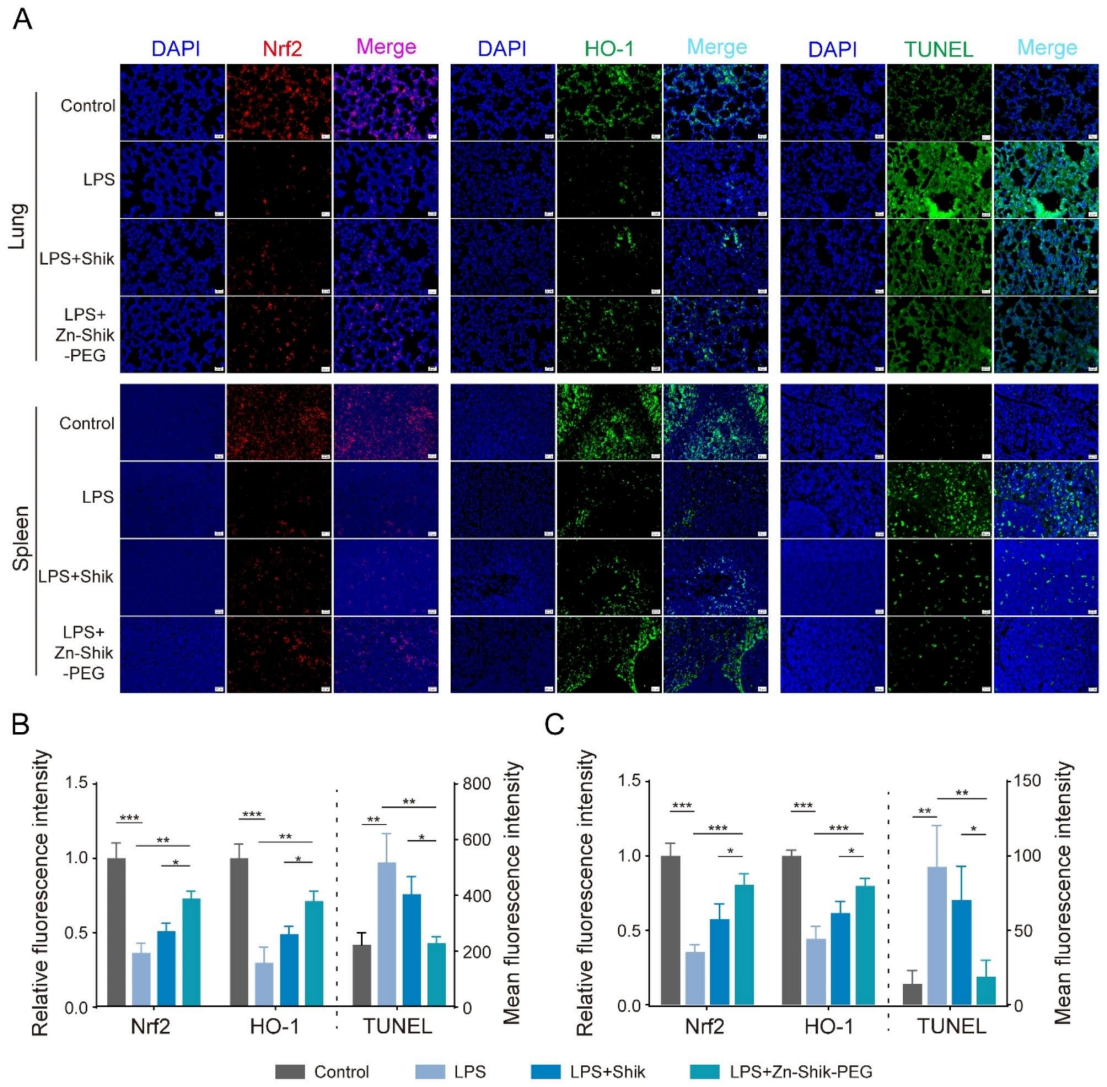
Mechanism of action of Zn-Shik-PEG NPs in vivo (**A**) Representative fluorescence images of Nrf2 (left), HO-1 (middle) expression and TUNEL staining (right) in lungs and spleen after different treatments. Scale bar: 20 μm. Relative fluorescence intensity of Nrf2, HO-1 and mean fluorescence intensity of Tunel in lung (**B**) and spleen (**C**). Data are presented as mean ± SD (n = 3). Statistical analysis: *p < 0.05, ** *p* < 0.01, and ***p < 0.001

Considering that Zn-Shik-PEG NPs have ROS scavenging and anti-apoptosis effects in vitro, we also investigated the role of Zn-Shik-PEG NPs in ROS and anti-apoptosis in vivo, by performing immunofluorescence staining of ROS-related factors Nrf2, HO-1 and TUNEL staining in lung and spleen tissues. Similar results were clearly found in mice tissues compared with in vitro results. As shown in Fig. 6, LPS decreased the expression of Nrf2 and HO-1, while Zn-Shik-PEG NPs could markedly reverse this trend. In addition, TUNEL staining showed a much lower number of apoptotic cells in the lungs and spleens of LPS/Zn-Shik-PEG-treated mice than in the LPS treated mice. These results suggest that Zn-Shik-PEG NPs possess significant ROS scavenging and anti-apoptosis capacity in both lung and spleen.

Finally, the in vivo biocompatibility of Zn-Shik-PEG NPs was evaluated (Figure S16). There were no abnormalities in blood routine data of healthy mice after Zn-Shik-PEG NPs treatment. In addition, there were no apparent histopathological abnormalities or lesions observed in the heart, liver, spleen, kidney and lung (Figure S16J), indicating that Zn-Shik-PEG NPs are virtually non-toxic to major organs. Collectively, these results demonstrated that the Zn-Shik-PEG NPs are highly biocompatible and their therapeutic effects for sepsis is effective and safe.

## Conclusion

In summary, we developed Zn-Shik-PEG NPs to compensate for the deficiency of low water solubility, low bioavailability and high toxicity of shikonin, and effectively alleviating systemic inflammation and multi-organ functional damage in sepsis. We found that Zn-Shik-PEG NPs could effectively improve the intracellular ROS clearance ability by regulating the Nrf2/HO-1 pathway, meanwhile Zn-Shik-PEG NPs could inhibit the inflammation and apoptosis mediated by intracellular NLRP3 inflammasome activation by regulating the AMPK/SIRT1 pathway (Scheme 1). As a result, Zn-Shik-PEG NPs significantly attenuated systemic inflammation response and protected against multiple organs injury in sepsis mice. Overall, Zn-Shik-PEG NPs may be a promising drug candidate for the treatment of sepsis.

## Experimental section

### Synthesis of Zn-Shik-PEG NPs

Shikonin ethanol solution (2 mL, 4 mg/mL) was added into ZnCl_2_ aqueous solution (20 mL, 2.5 mg/mL) and then stirred for 1 h at room temperature. The resulting Zn-Shik nanoparticles were centrifuged for 10 min. Subsequently, 1 mL NH_2_-mPEG solution (50 mg) was added into Zn-Shik ethanol solution (10 mL) under stirring at room temperature for 24 h. The resulting solution was transferred to a rotary evaporator to remove the ethanol and resuspended in 5 mL of DI water for further use.

### Cell culture

RAW264.7 mouse macrophages were seeded in DMEM medium supplemented with FBS (10%), penicillin (100 U/mL) and streptomycin (100 mg/mL), meanwhile placed in a 37℃, 5% CO_2_ strictly sterile incubator.

### Fluorescence assay of ROS level

The ROS level was tested by DCFH-DA ROS assay kit. RAW264.7 cells were inoculated into a 6-well plate (5 × 10^6^ cells/well) and incubated overnight. Cells were pretreated with Zn-Shik-PEG NPs (5 µg/mL) and Shikonin for 30 min and then incubated with LPS (0.1 µg/mL) for 24 h. DCFH-DA was then diluted with serum-free medium to a final concentration of 10 µM and added to per well. After collecting the cells and washing them twice with PBS, the fluorescent images were captured under a Leica fluorescence microscope.

### Western blot analysis

Cells were pretreated with Zn-Shik-PEG NPs (5 µg/mL), Shikonin and BML-275 (100 µM) for 0.5 h and then incubated with LPS (0.1 µg/mL) for 24 h. Then, 100 µL of RIPA lysate containing PMSF (PMSF: RIPA = 1: 100) was added to per well, the cells were fully lysed on ice and the cell proteins were extracted. The protein concentration was quantified with Bradford protein kit, then the samples were mixed with loading buffer and heated at 95 °C for 10 min. Protein samples were separated in SDS-PAGE and transferred to PVDF membrane that was blocked with 5% skimmed milk. Next, membranes were incubated with primary antibody overnight. After three washes in TBST, the membranes were incubated with appropriately diluted secondary antibodies at room temperature prior to visualization using ChemiDoc XRS imaging system.

### Cell immunofluorescence

RAW264.7 cells were inoculated into six-well plate with cell climbing slice (24 mm) at a density of 5 × 10^6^ cells/well and incubated overnight. Following NPs or shikonin treatment, cells were fixed with 4% paraformaldehyde in PBS for 20 min, and permeabilized with 0.5% Triton X-100 for 20 min. After three washes in PBS, they were incubated with normal goat serum for 30 min at room temperature. Afterwards, the diluted primary antibody was dripped on the climbing slices and completely covered the cells, and incubated at 4℃ overnight, followed by incubation with fluorescent secondary antibody 488 or 647. Finally, the cell climbing slices were sealed with antifade mounting medium (including DAPI). The images were captured by fluorescence microscope and analyzed by Image J software.

### Establishment of animal models and treatment

All mice were arbitrarily segregated into blank group, LPS group, shikonin group and Zn-Shik-PEG NPs group. Shikonin and Zn-Shik-PEG NPs were intraperitoneally injected 1 h before LPS treatment. After the successful establishment of the model, the survival rate, clinical score and body temperature of some mice within 72 h were closely monitored and recorded. Another part of mice were killed 24 h after the model was successfully established, and their lungs, livers, kidneys, spleens and hearts were taken for H&E staining, TUNEL staining, immunofluorescence staining and part of lung tissues was used to measure lung index and MPO. The serum was collected and stored for the detection of the expression of inflammatory factors IL-6, TNF-α and other biochemical indicators.

### CRE, BUN, ALT, AST detection

Serum CRE, BUN, ALT, AST were measured strictly in accordance with the product instructions. After reading OD, the expression of CRE, BUN, ALT, AST in each serum was calculated according to the corresponding formula.

### Measurement of TNF-α and IL-6

Prepared sample, standard substance and biotin antigen were added in the 96-well plate, reacting at 37℃ for 30 min. And then added avidin-HRP reacting at 37℃ for 30 min after washing the plate for 5 times. Next, TMB chromogen solution was added, developing at 37℃ for 10 min. Finally, added termination liquid and took the OD value within 10 min. The expression of TNF-α and IL-6 in each serum were calculated by logistic curve (four parameters).

### Statistical analysis

Statistical analysis was carried out with SPSS 26.0 software, and measurement data were presented as mean ± SD. One-way analysis of variance (ANOVA) was used for comparison between groups when data met normal distribution and homogeneity of variance, and the least significant difference (LSD) was used for further pairwise comparisons. *P* < 0.05 was considered as the criterion for statistically significant differences.

### Electronic supplementary material

Below is the link to the electronic supplementary material.

Supplementary Material 1

## Data Availability

The data that support the findings of this study are available from the corresponding author upon reasonable request.
