# Supplementary material for: Zn-Shik-PEG nanoparticles alleviate inflammation and multi-organ damage in sepsis
Source: J Nanobiotechnology. 2023 Nov 25;21:448. doi: 10.1186/s12951-023-02224-3 (PMC10675904; doi:10.1186/s12951-023-02224-3)
Supplement: Supplementary file 1 — Supplementary Material 1 [file 12951_2023_2224_MOESM1_ESM.docx]

Supplementary Material

Zn-Shik-PEG Nanoparticles Alleviate Inflammation and Multi-Organ Damage in Sepsis

Jie Guo, Yuqing Miao,* Fayi Nie, Fei Gao, Hua Li, Yuan Wang, Qi Liu, Tingbin Zhang, Xiaohang Yang, Li Liu, Haiming Fan, Qiang Wang,* and Haifa Qiao,*

Experimental Section

*Materials:* ZnCl_2_ was obtained from Tianjin Beilian Chemical Reagen Co., Ltd. NH_2_-mPEG (MW:2000) was purchased from Shanghai Tuoyang Biotechnology Co., Ltd. LPS (DH183-1) was purchased from Beijing Dingguo Changsheng Biotechnology Co. Ltd. Shikonin (S115193) was purchased from Aladdin Company (Shanghai, China). BML-275 (S7306) was purchased from Selleck (Texas, USA). TRIzol Reagent (15596-026) was purchased from Invitrogen (Vienna, Austria). DMEM high-glucose liquid culture medium (PYG0096) was purchased from Boster (Hubei, China). Fetal bovine serum (FBS) (04-001-1A) was purchased from Bioind (Beit Haemek, Israel). 0.25% Trypsin-EDTA (25200-056) was purchased from Gibco Laboratories (Grand Island, NY, USA). ROS (E004-1-1), MPO (A044-1-1), CRE (C011-2-1), BUN (C013-2-1), ALT (C009-2-1) and AST (C010-2-1) was purchased from Naniing Jiancheng Bioengineering Institute (Jiangsu, China). Antibodies against Nrf2 (16396-1-AP) and HO-1 (10701-1-AP) were obtained from Proteintech (Hubei, China). CCK8 (ab228554), IL-6 (ab222503), TNF-α (ab208348) and ELISA kits Antibodies against AMPK (ab32047), p-AMPK (ab133448), SIRT1 (ab110304), NLRP3 (ab270449), ASC (ab307560), IL-1β (ab254360), IL-18 (ab207323) and β-actin (ab8226) were obtained from Abcam (Cambridge, MA, USA). Antibodies against Caspase-1 (#24232) and Cleaved-Caspase-1 (#89332) were obtained from Cell Signaling Technology (Beverly, MA, USA). One-step TUNEL In Situ Apoptosis Kit (E-CK-A321) were obtained from Elabscience (Hubei, China).

*Characterizations:* Transmission electron microscope (TEM) was conducted using a transmission electron microscope (JEOL, JEM-1400 Flash). The hydrodynamic diameters of the samples were evaluated with dynamic light scattering (DLS, Malvern Zetasizer nano-ZS instrument). XPS measurements were performed by X-ray photoelectron spectroscopy (XPS, Thermo Scientific Nexsa). The ultraviolet–visible (UV-Vis) absorption spectra were carried out on a Nano Drop one. The Fourier transform infrared spectrum was collected with a Tensor 27 IR spectrometer (Bruker, Germany). Fluorescence images were captured by fluorescence microscope (Leica, Germany).

*The cell counting kit-8 (CCK-8) assay:* RAW264.7 cell suspension was inoculated into a 96-well plate, with 100 μL (5000 cells per well) in per well, blank holes (only containing culture medium) were set at the same time, and then the culture plate was cultured in an incubator for 24 hours. Except for the control well and blank well, the cells were pretreated with Zn-Shik-PEG NPs (2 μg/mL, 5 μg/mL and 10 μg/mL) and Shikonin (1 μg/mL, 3 μg/mL and 5 μg/mL) respectively, and then incubated with LPS (0.1 μg/mL) for 24 h. Afterwards, CCK-8 solution (10 μL) was added to each well and incubated at 37°C for 4 hours. The OD value of per well at 450 nm (As: test well, Ac: control well, Ab: blank well) was measured by enzyme-labeled instrument, and the cell viability was calculated according to the following formula. Cell viability (%) = [(As-Ab)/(Ac-Ab)] × 100%.

*Real-Time Polymerase Chain Reaction (RT-PCR)*: TRIzol was used to extract RNA, and the quality and the total concentration of RNA were evaluated by spectrophotometer. The absorbance ratio of 260/280 nm was 1.8~2.2, which indicated that there were pure RNA samples. The cDNA was synthesized by reverse transcription according to the instructions of the kit, and PCR amplification was carried out according to the amplification kit. The target mRNAs were quantified by real-time PCR using SYBR Green master mix and the relative quantitative analysis of IL-6 and TNF-α was carried out by using 2^‐ΔΔCt^ method with GAPDH as internal reference. Primers used for the experiment were synthesized by Tsingke Biotechnology Co., Ltd. (Table 1).

**Table S1.** Primers of housekeeping gene GAPDH and targeted genes

| Target gene | Primer pairs (5'→3') |
| --- | --- |
| IL-6 | 5'-GGGACTGATGCTGGTGAC-3' |
|  | 5'-AAGCCTCCGACTTGCGA-3' |
| TNF-α | 5'-CGATGGGTTGTACCTT-3' |
|  | 5'-TACTTGGGCAGATTGAC-3' |
| GAPDH | 5'-TCAACGGCACAGTCAAG-3' |
|  | 5'-CTCGCTCCTGGAAGAT-3' |

*Cell TUNEL staining:* After treatment with NPs, cells were rinsed with PBS, fixed with 4% paraformaldehyde, permeabilized with 0.2% Triton X-100 at 37°C for 10 min. Then immersed samples in PBS and rinse them for 3 times. Thereafter, TdT equilibration buffer was added and reacted at 37°C for 30 min. After absorbing TdT equilibration buffer with absorbent paper, each sample was dripped with 50 μL labeled working solution, and then put into a wet box to react at 37°C for 2 h in the dark. The samples were rinsed in PBS for three times, then DAPI solution was added, and incubated in a dark environment for 5 min. Finally, the cell climbing slices were sealed with antifade mounting medium. The images were observed by fluorescence microscope and analyzed by Image J software.

*Animal experiments:* The healthy male KM mice (24±2 g) were purchased from the Experimental Animal Center of Xi'an Jiaotong University. Mice were maintained in standard rodent microisolation cages with filter caps at the SPF experimental center with 2-4 animals per cage. And placed in a stable 12/12 h light/dark cycle room with temperature (22±2℃) and humidity (55±5%), supply sufficient sterilized granular rat food and sterile drinking water, move freely, and replace the padding to maintain hygiene. All animal procedures were performed in accordance with the Institutional Animal Care and Use Committee. The animal experiments were approved by the Experimental Animal Ethics Committee of Shaanxi University of Chinese Medicine. During the course of the experiment, the operation of animals complies with the relevant provisions of the Guiding Opinions on the Good Treatment of Laboratory Animals to minimize the pain of animals. For cecal ligation and puncture (CLP): Mice were randomly divided into sham operation group (sham), CLP group, Shikonin group (5 mg/kg) and Zn-Shik-PEG NPs group (10 mg/kg). Shikonin and Zn-Shik-PEG NPs were injected intraperitoneally one hour before CLP operation.

*CLP-induced sepsis model mice:* Briefly, the mice fasted for 12 hours before operation, and were free to take water. After anesthesia with the breathing anesthesia machine of small animals, the animals were placed in supine position on a heating pad at 37℃. After sterilization, an abdominal incision (1 cm) was made from the midline of the abdomen to expose the cecum, which was subsequently ligated with surgical thread (4-0). Then, gently put the cecum back into the abdominal cavity after puncturing the cecum stump with No.21 needle for two times. Finally, sutured the abdominal incision with surgical thread (4-0). The sham group underwent the same procedure without ligation or perforation.

*Survival rate and clinical score:* After the successful establishment of the model, the survival time and living status of mice in each group were closely observed within 72 hours. The death time and the number of mice were recorded. The mice were scored every 12 hours according to the following scoring standards (Table 2), and when the score of the mice was 5, they were killed humanely.

**Table S2.** Clinical scoring standard

| **Score** | **Symptom** |
| --- | --- |
| 0 | No symptoms |
| 1 | Piloerection and huddling |
| 2 | Piloerection, huddling, and diarrhea |
| 3 | Lack of interest in surroundings and severe diarrhea |
| 4 | Decreased movement and listless appearance |
| 5 | Loss of selfrighting reflex |

*Lung Index:* The lung tissues were obtained and weighed, and the lung index of the mice was calculated as: Lung index (%) = lung weight (g)/body weight (g) ×100.

*MPO detection in lung tissues:* Lung tissues were weighed, and added homogenate medium according to the weight-volume ratio of 1:19 to prepare 5% tissue homogenate, then added different reagents in turn according to the operation instructions, and measured the light absorption value of per well at 460 nm with spectrophotometer.

*Pathological analysis of tissues:* Tissues of heart, liver, spleen, lung and kidney, which had been fixed in 4% paraformaldehyde for 48 h were removed and embedded in paraffin. Tissue sections (5 μm) were stained with hematoxylin-eosin and dehydrated with an ethanol gradient, xylene transparent twice and sealed with neutral resin. The morphology and structure were of tissues were observed under a microscope. The pathological analysis and inflammation scoring performed by a pathologist used a semiquantitative method [1, 2]. Inflammation Scoring was based on number of inflammatory cells per high-power field (score 0: 0; score 1: 1–25, score 2: 26–50; score 3: >50).

*Tissue immunofluorescence:* After the sections were repaired with sodium citrate, they were incubated with normal goat serum containing 0.3% Triton X-100 at 37℃ for 30 min, and then spun-dry without washing. The sections were incubated with primary antibody overnight at 4℃, and followed by incubation with fluorescent secondary antibody 488 or 647 nm at room temperature for 1 h in the dark. Finally, the sections were sealed with antifade mounting medium (including DAPI). The images were observed by fluorescence microscope and analyzed by Image J software.

*Tissue TUNEL staining:* Paraffin sections were removed, and the sections were rinsed in PBS for 3 times after dewaxing and hydration. The water on the samples were absorbed with filter paper. And 100 μL 1× protease K working solution were dropped on each samples, reacting at 37°C for 20 min. Then immersed sections in PBS and rinsed them for 3 times. 100 μL TdT equilibration buffer was added to each sample, and the reaction was carried out at 37°C for 30 min. After absorbing TdT equilibration buffer with absorbent paper, each sample was dripped with 50 μL labeled working solution, and then put into a wet box to react at 37°C for 2 h in the dark. The samples were rinsed in PBS for three times, then DAPI working solution was added, and incubated for 5 min at room temperature in the dark. Finally, the cell climbing slices were sealed with antifade mounting medium. The images were observed by fluorescence microscope and analyzed by Image J software.

*Routine blood test:* Blood was collected from mouse orbit and dripped into anticoagulation tubes, mixing thoroughly. To detect white blood cell (WBC), lymphocyte (LYM), platelet (PLT), red blood cell (RBC), hemoglobin (HGB), hematocrit (HCT), mean corpuscular volume (MCV), mean corpuscular hemoglobin (MCH) and mean corpuscular hemoglobin concentration (MCHC) by an auto hematology analyzer (Shinova, Shenzhen, China).

References

[1] Guenthart, B. A.; O’Neill, J. D.; Kim, J.; Queen, D.; Chicotka, S.; Fung, K.; Simpson, M.; Donocoff, R.; Salna, M.; Marboe, C. C.; et al. Regeneration of severely damaged lungs using an interventional cross-circulation platform. Nat. Comm. 2019, 10, 1985.

[2] Huang, S. C.; Wu, J. F.; Saovieng, S.; Chien, W. H.; Hsu, M. F.; Li, X. F.; Lee, S. D.; Huang, C. Y.; Huang, C. Y.; Kuo, C. H. Doxorubicin inhibits muscle inflammation after eccentric exercise. J Cachexia Sarcopenia Muscle 2016, 8, 277-284.

**
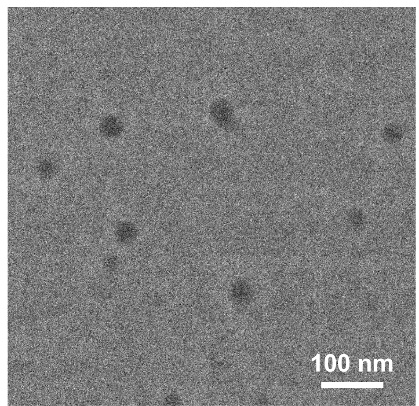
**

**Figure S1.** TEM images of Zn-Shik NPs


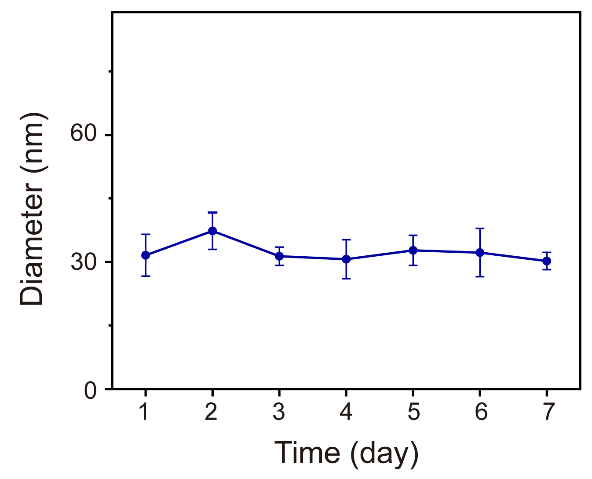


**Figure S2.** Colloidal stability of the Zn-Shik-PEG NPs.

**Table S3.** FTIR characteristic bands of Zn-Shik-PEG

| Wavenumber (cm^-1^) | Assignment |
| --- | --- |
| 1607, 1550 | C=C aromatic ring stretching band |
| 1430 | -OH bend |
| 1350 | Bending vibration of methylene group |
| 1275 | HO-C stretching |
| 1104 | C-N stretching vibration |
| 1064 | C-O stretch |


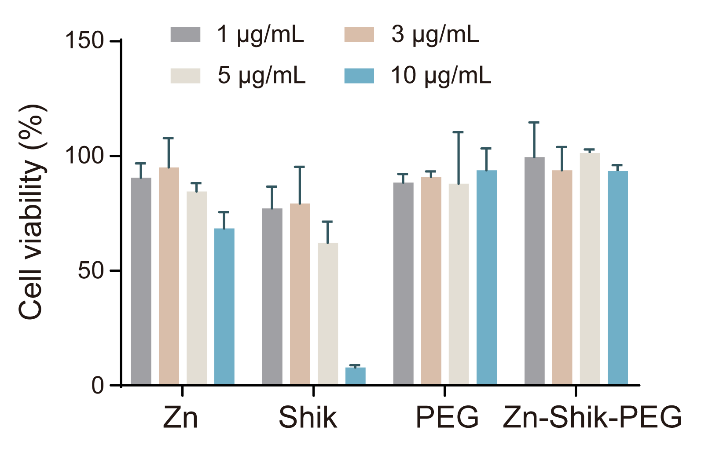


**Figure S3.** Cytotoxicity of Zn, Shikonin, PEG and Zn-Shik-PEG to RAW 264.7 cells.


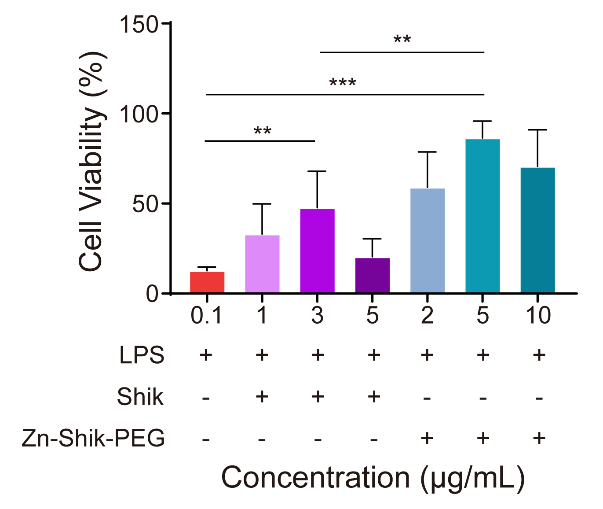


**Figure S4.** Cell viability (CCK-8) of RAW264.7 cells incubated with different doses of shikonin and Zn-Shik-PEG NPs for 24 h.


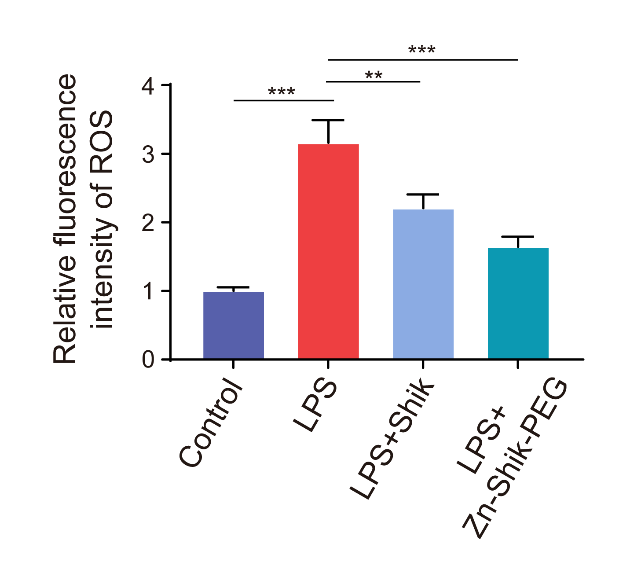


**Figure S5.** Quantitative analysis of the relative fluorescence intensity of ROS.


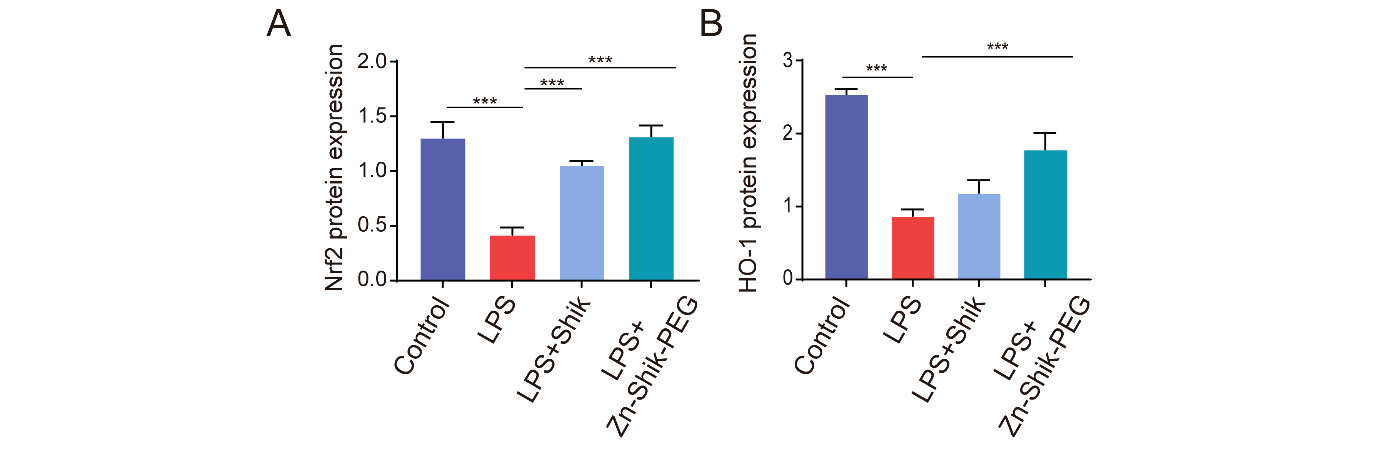


**Figure S6.** Quantitative analysis of Nrf2, HO-1 expression levels.


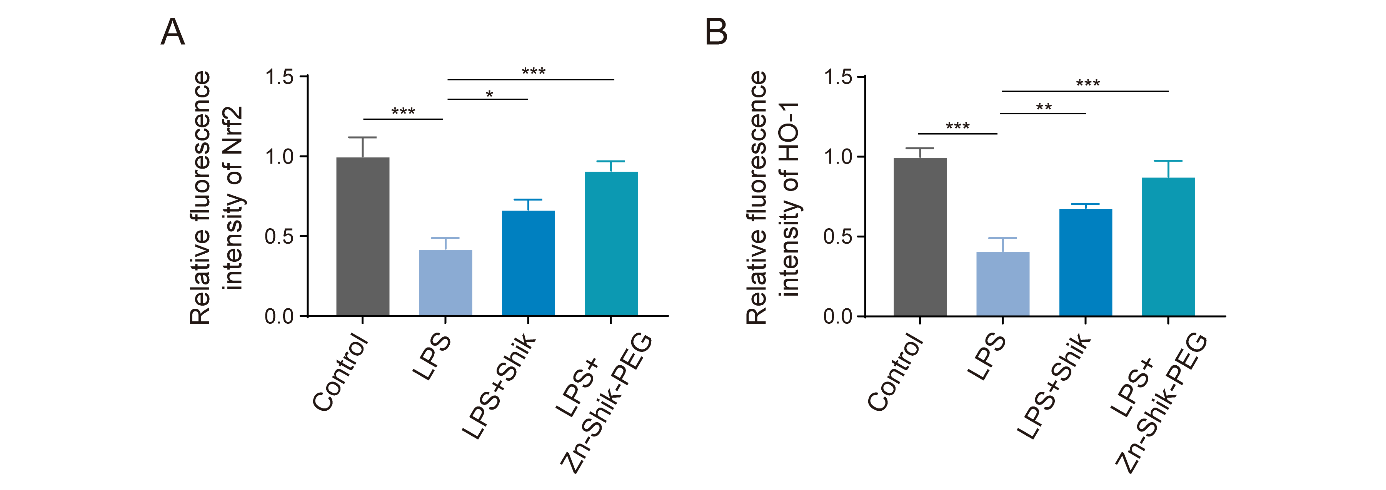


**Figure S7.** Relative fluorescence intensity of Nfr2 (A) and HO-1 (B). Data are presented as mean ± SD (n = 3). *p <0.05, ** p <0.01, and ***p <0.001.


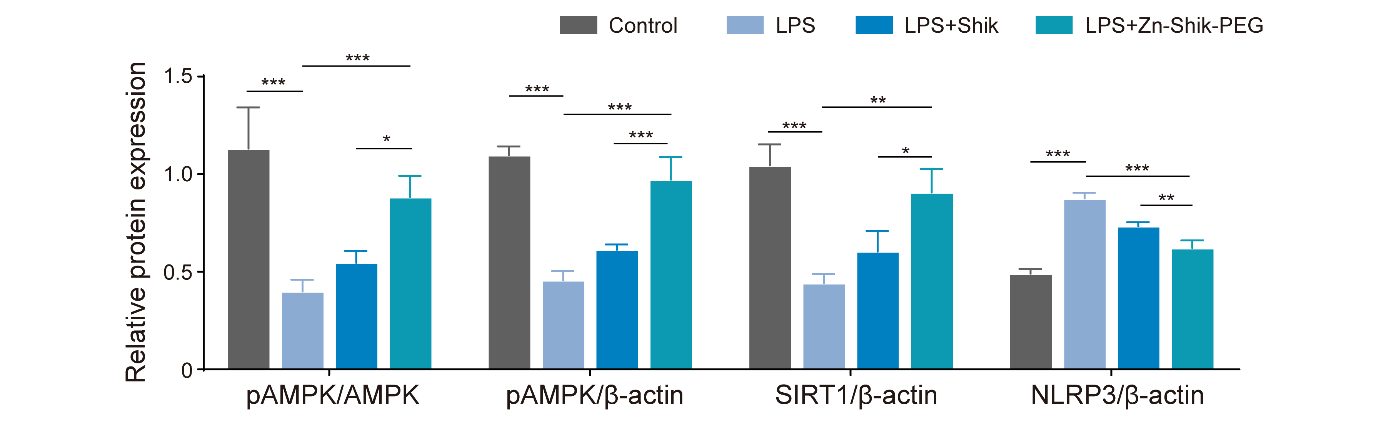


**Figure S8.** Quantitative analysis of AMPK, p-AMPK, SIRT1, NLRP3 expression levels.


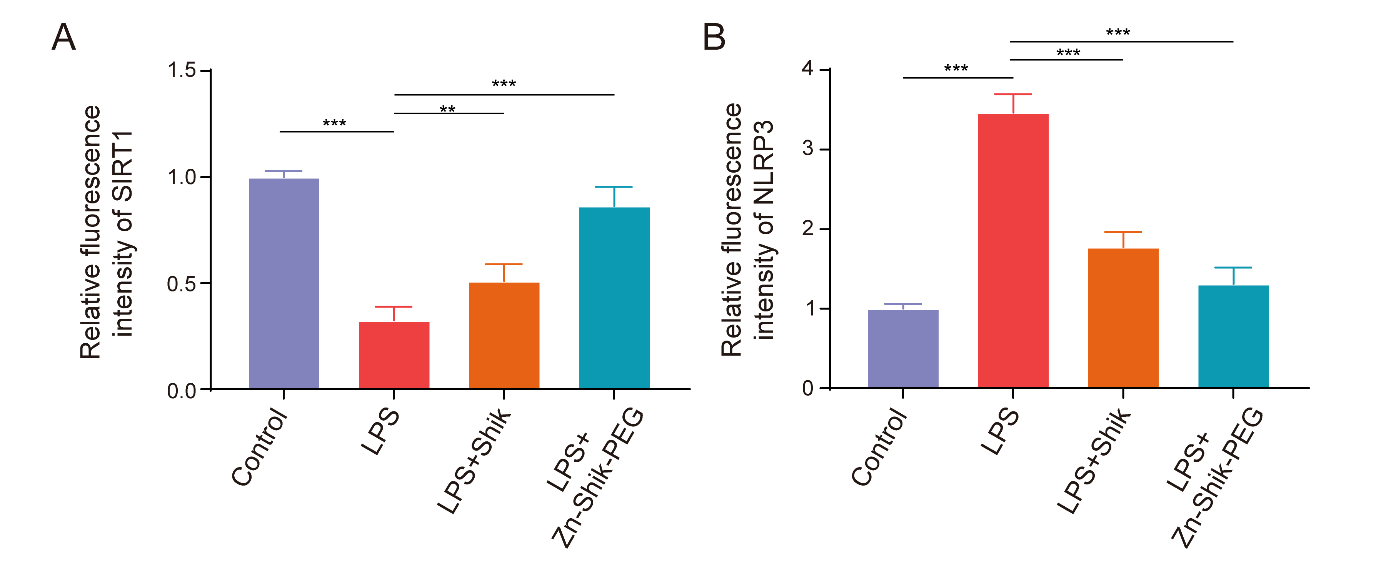


**Figure S9.** Relative fluorescence intensity of SIRT1 (A) and NLRP 3 (B).


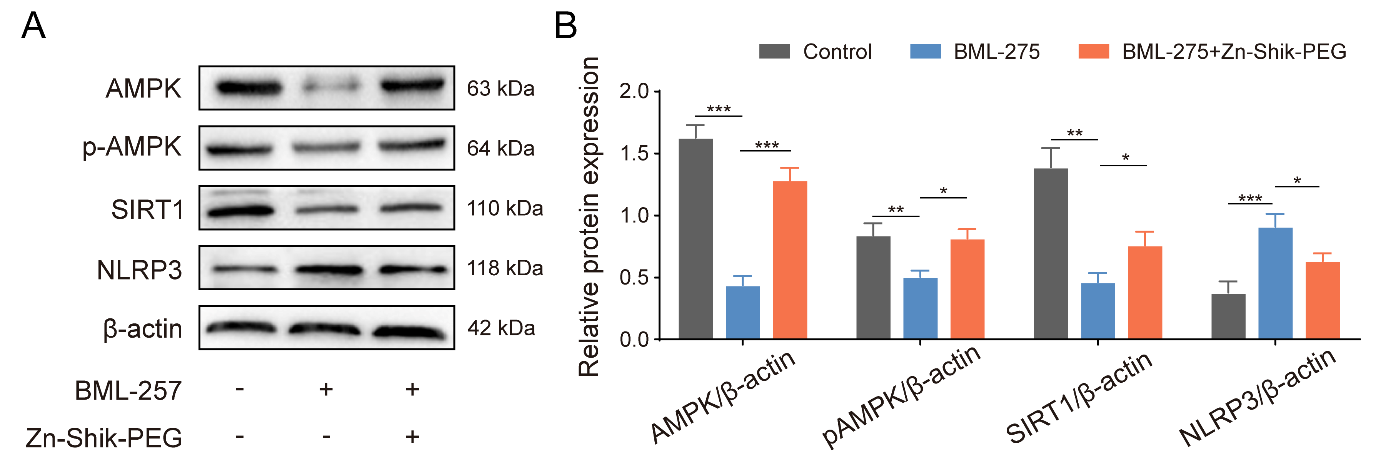


**Figure S10.** Representative western blotting bands of AMPK, p-AMPK, SIRT1, NLRP3 and quantitative analysis of their expression levels after addition of the inhibitor BML-275. Data are presented as mean ± SD (n = 3). Statistical analysis: *p < 0.05, ** p < 0.01, and ***p < 0.001.


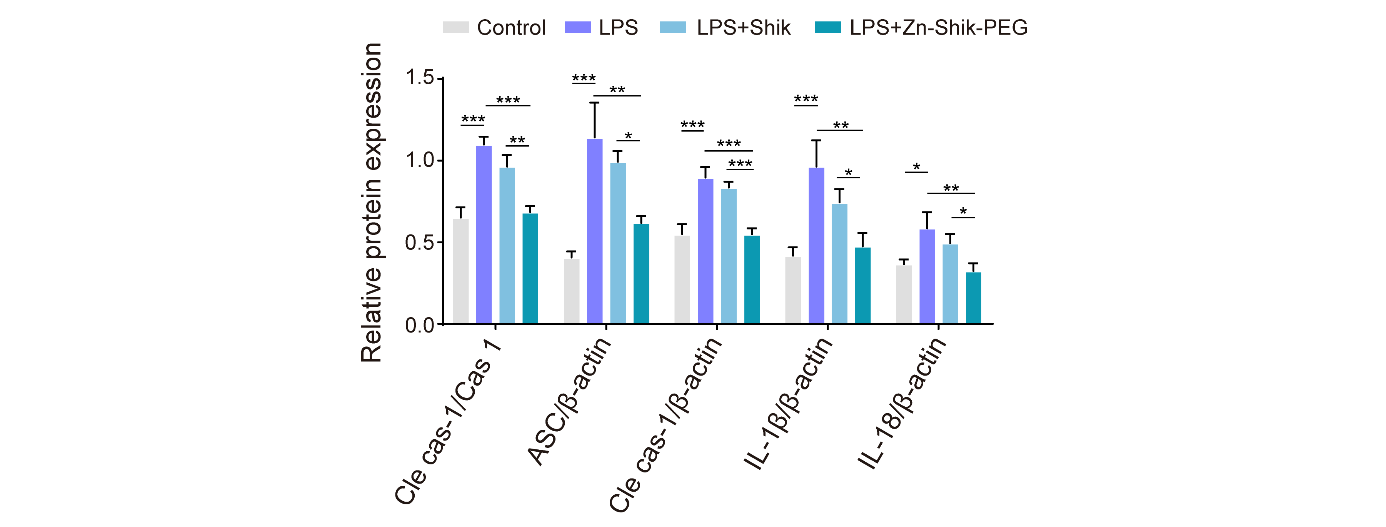


**Figure S11.** Quantitative analysis of ASC, cleaved caspase-1, caspase-1, IL-1β, IL-18 expression levels


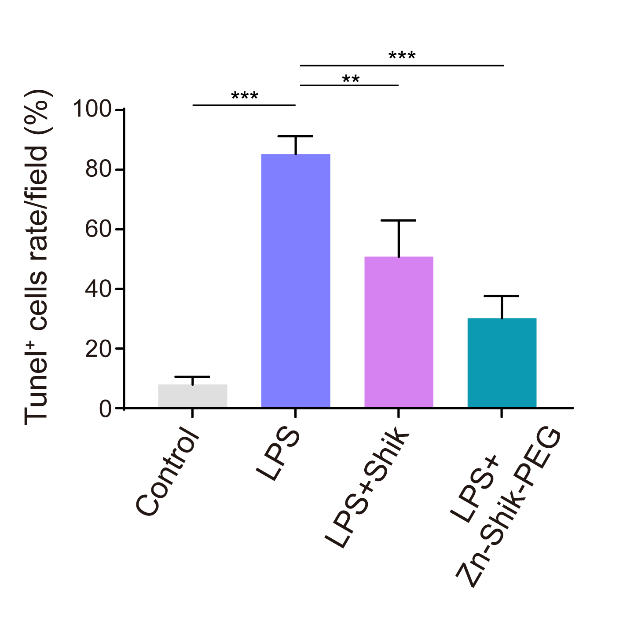


**Figure S12.** Quantitative analysis of TUNEL staining. Data are presented as mean ± SD (n = 3). Statistical analysis: ** p < 0.01, and ***p < 0.001.


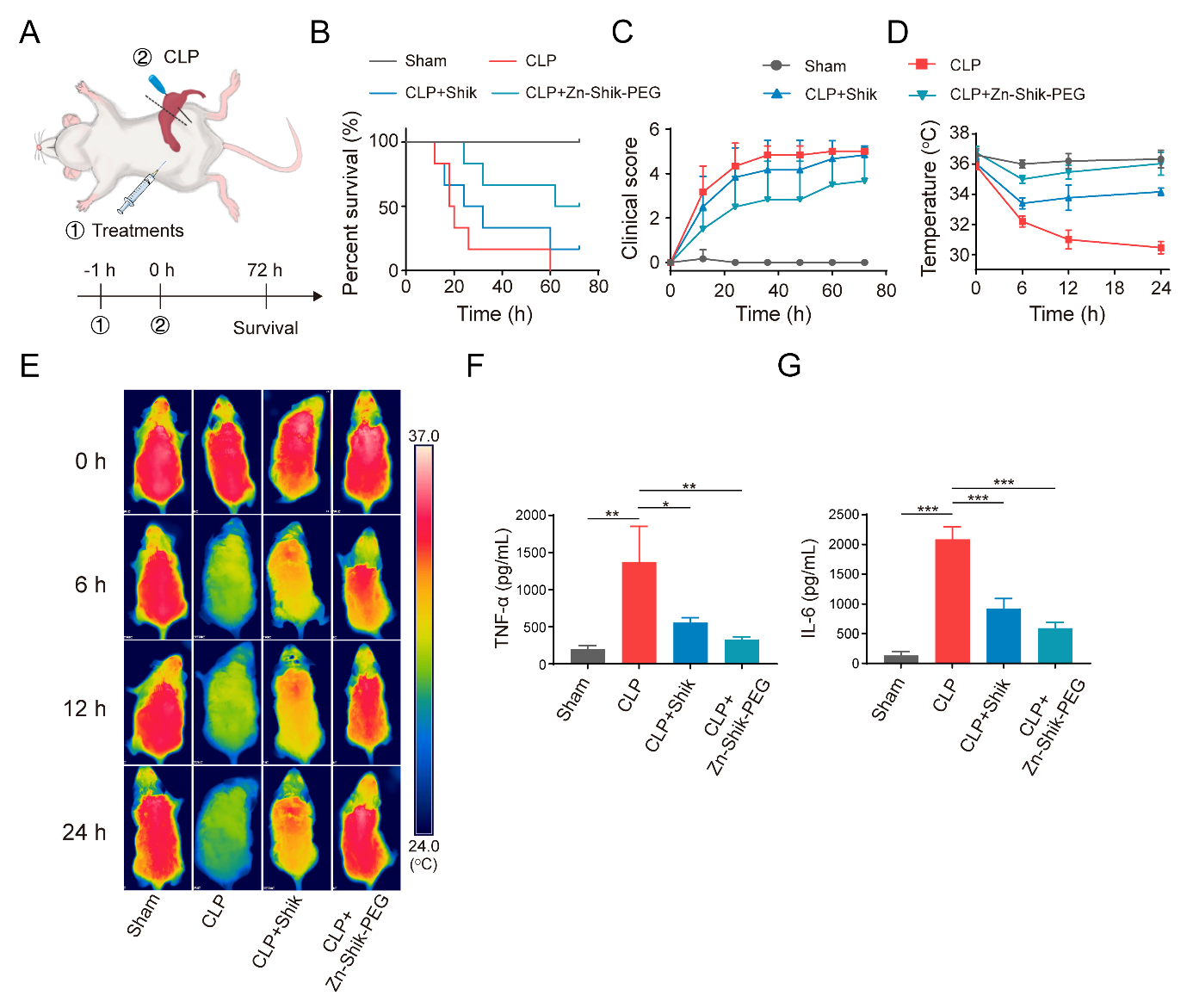


**Figure S13.** Therapeutic efficacy of the Zn-Shik-PEG NPs against CLP-induced sepsis in mice. (A) Experimental procedures for the CLP. (B, C) Survival and clinical score analysis of the mice. (D, E) Body temperature change and thermographic images of mice in different treatment groups. (F, G) The level of pro-inflammatory cytokine TNF-α (F) and IL-6 (G) in the serum of septic mice.


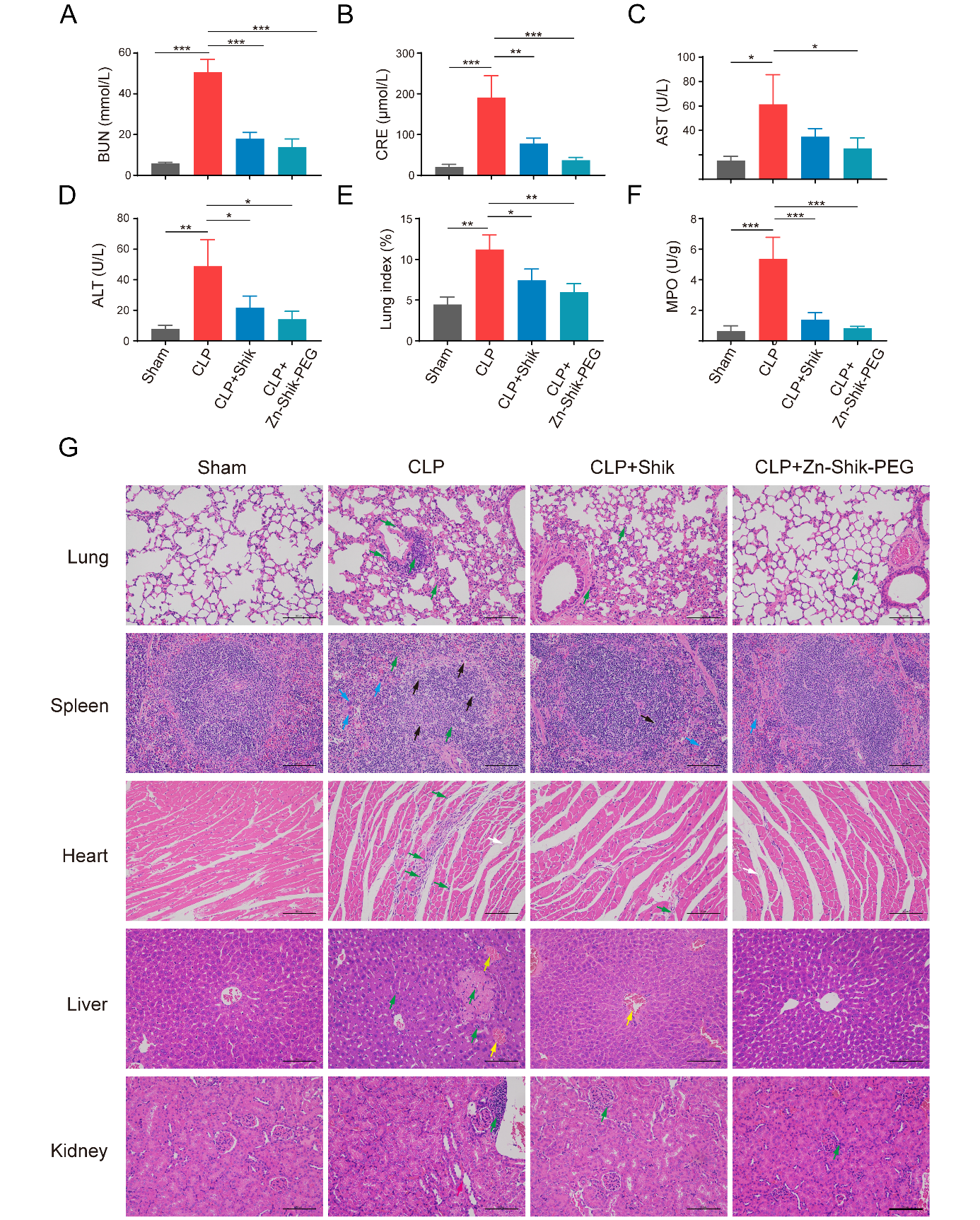


**Figure S14.** Protective effect of Zn-Shik-PEG NPs on CLP-induced multi-organ damage. (A-D) BUN, CRE, AST, ALT, levels in serum were detected by ELISA kits. (E) Lung index in each treatment group. (F) MPO activity was measured in lungs after CLP challenge. (G) H&E staining of lung, spleen, heart, liver, kidney tissue. Green arrows indicate inflammatory cell infiltration; black arrows indicate lymphocyte necrosis; blue arrows indicate megakaryocytes; white arrows indicate collagen fibers between cardiac muscle fibers; yellow arrows indicate vascular congestion; pink arrow indicates tubular epithelial cell degeneration. Scale bar: 100 μm. Data are presented as mean ± SD (n = 3). *p <0.05, **p <0.01, and ***p <0.001.


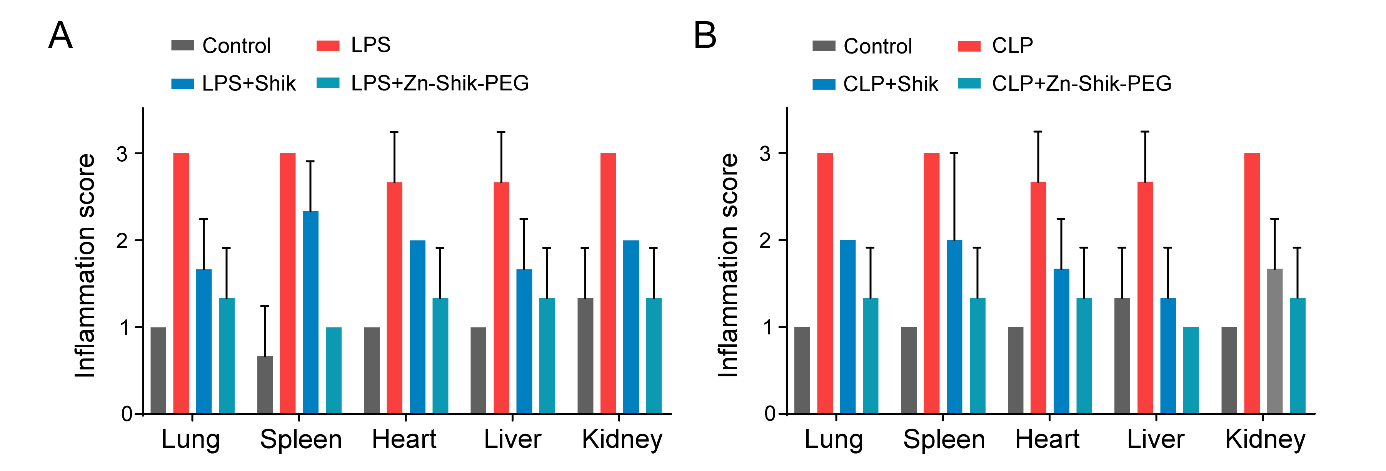


**Figure S15.** Semiquantitative analysis of tissues based on haematoxylin and eosin (H&E) staining by inflammation score. (A) LPS model, (B) CLP model.


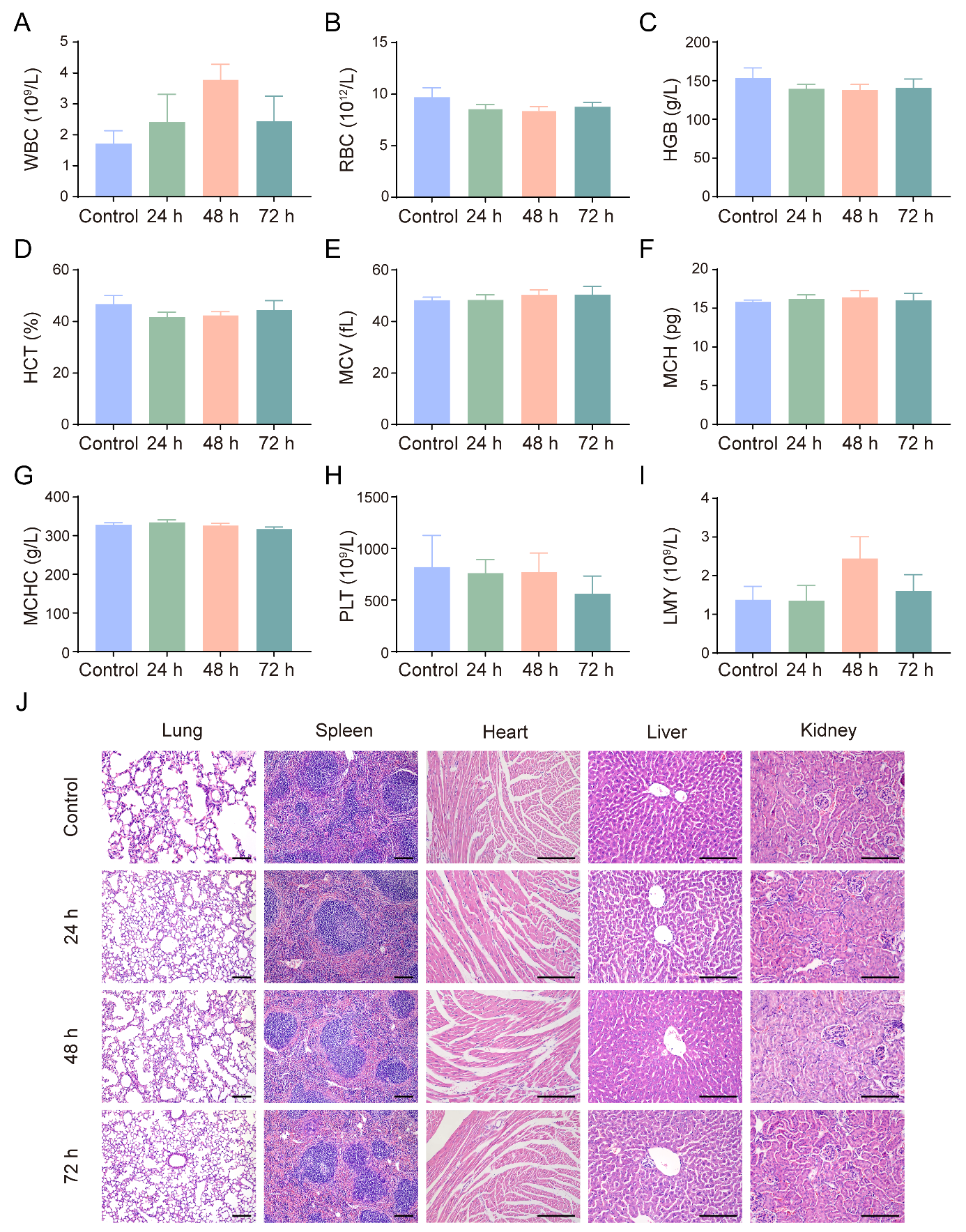


**Figure S16.** Toxicity of Zn-Shik-PEG NPs in vivo. (A-I) Blood panel data of normal mice (Control) and mice post Zn-Shik-PEG NPs injection at different time points (24, 48, 72 h). (J) H&E staining of lung, spleen, heart, liver, kidney after Zn-Shik-PEG NPs treatment at different time points (24, 48, 72 h). Scale bar: 50 μm.
